# Supplementary material for: Structural roles of guide RNAs in the nuclease activity of Cas9 endonuclease
Source: Nat Commun. 2016 Nov 2;7:13350. doi: 10.1038/ncomms13350 (PMC5097132; doi:10.1038/ncomms13350)
Supplement: Supplementary Information — Supplementary Figures 1-8, Supplementary Table 1 and Supplementary references. [file ncomms13350-s1.pdf]

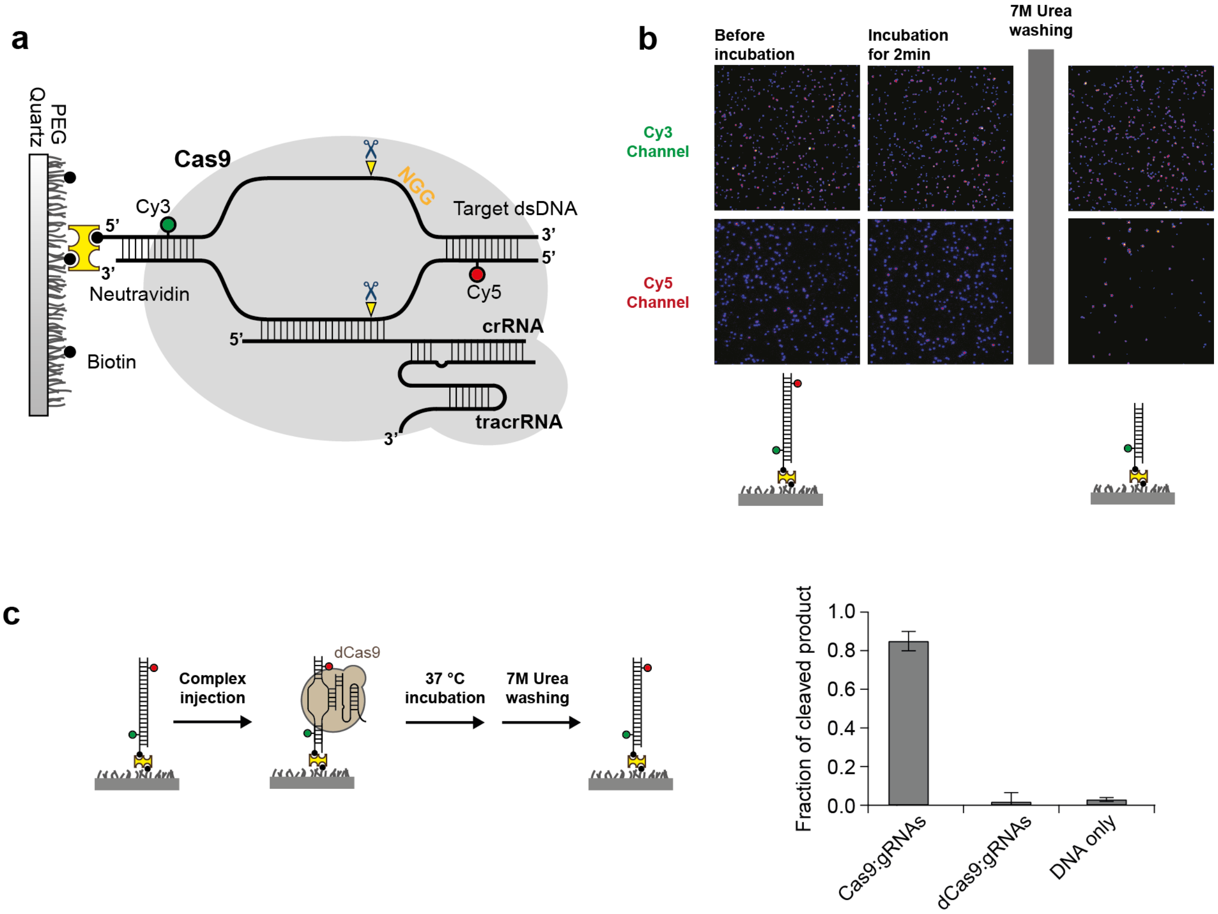

Supplementary Figure 1. Ratiometric fluorescence visualization of DNA cleavage by Cas9:gRNA. (a) A dsDNA labeled by Cy3 and Cy5 with an inter-probe distance of > 30 bp was tethered to a PEG-coated surface via biotin-neutravidin. When Cas9:gRNA was added, it recognized and bound to the targeted sites via Watson–Crick base-pairing. (b) Schematics and single-molecule surface fluorescence images of target DNA before (left) and after (right) the degradation caused by the nuclease activity of Cas9:gRNA. The middle panel shows DNA-bound Cas9:gRNA on the surface before the liberation of Cas9:gRNA by 7 M urea denaturation. (c) To verify the effect of 7M urea solution on dsDNA or biotin-neutravidin denaturation after RNA-DNA heteroduplex was formed with dCas9, we performed the same cleavage assay with dCas9, as a control. The result clearly shows that no significant loss of Cy5 molecules was observed.

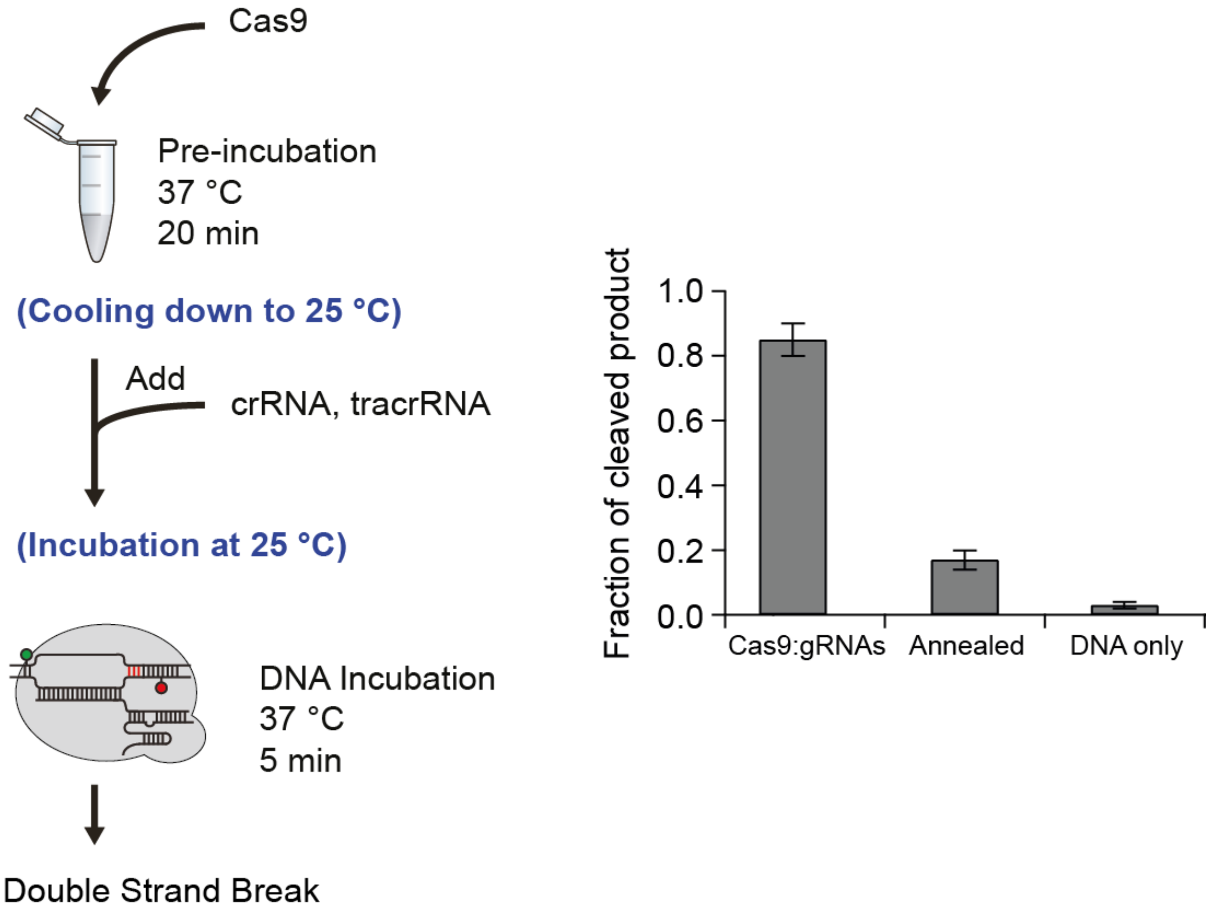

Supplementary Figure 2. Cleavage assay with the annealed Cas9. Procedural sequence to produce annealed Cas9 complex (left) and DNA cleavage efficiency with the annealed Cas9 (right). The reversible conformational change of structurally inactive Cas9 into activated Cas9 is not allowed when the thermal energy is not sufficient.

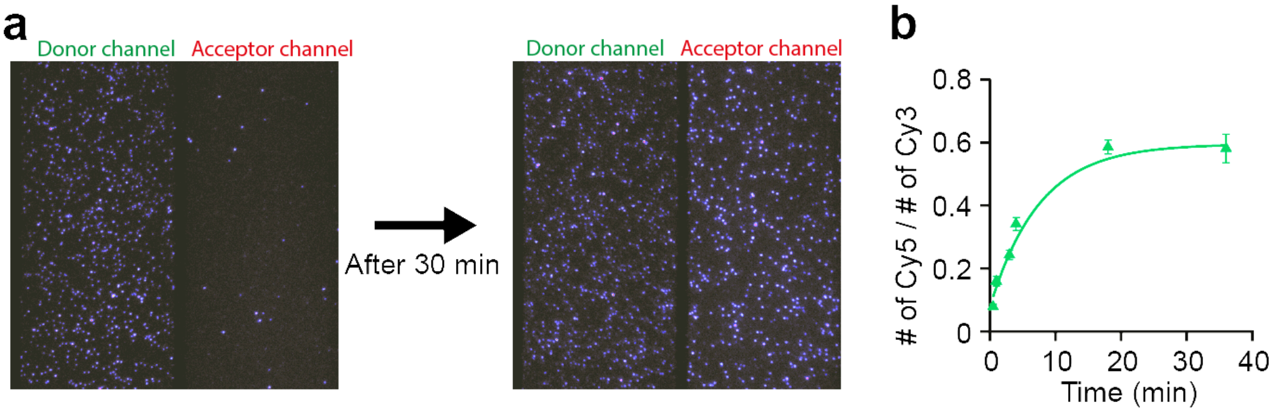

Supplementary Figure 3. Real-time imaging of Cas9:gRNA binding on target DNA. (a) Binding events of Cas9:gRNA on tethered DNA monitored by single-molecule surface image taken a few seconds after the injection of Cas9:gRNA (left) and after 37 ºC incubation for 30 min (right). (b) The plot of binding ratio vs. time for Cas9:gRNA.

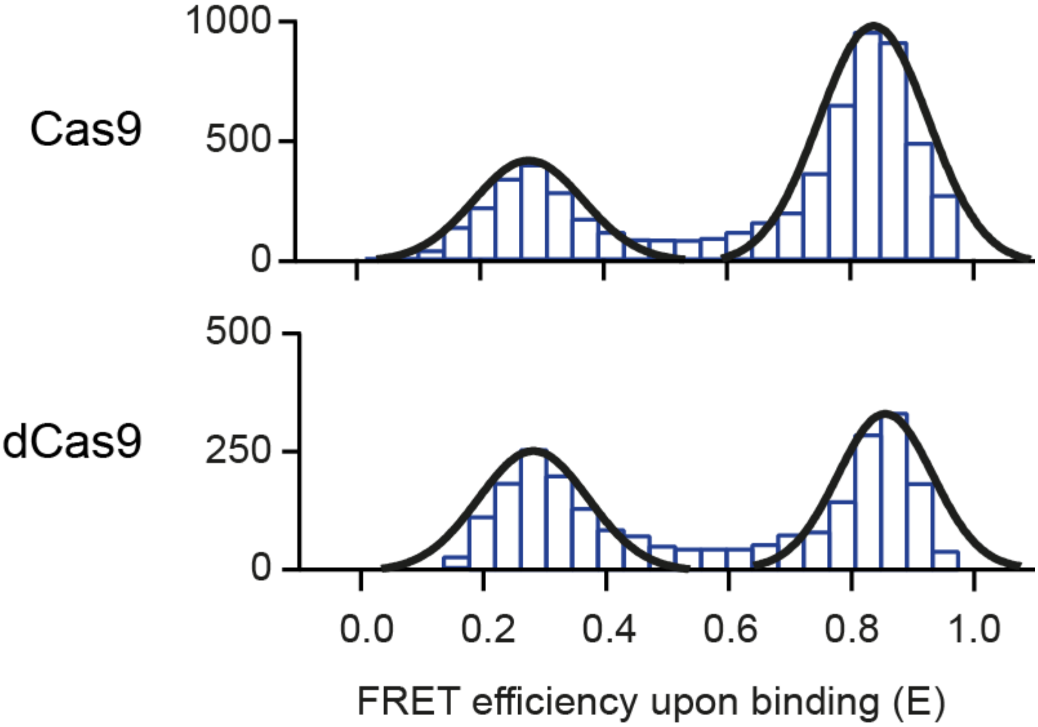

Supplementary Figure 4. The FRET efficiency histogram of dCas9:gRNA (dCas9 = "dead" Cas9, a catalytically inactive Cas9 by D10A and H840A mutations). When we replaced Cas9 with dCas9, nearly identical FRET states (E = 0.27 and 0.82) were observed, suggesting that neither FRET state is related to the cleaved product. Rather, these changes in the FRET efficiency are due to the structural flexibility associated with an intermediate state prior to the DNA cleavage.

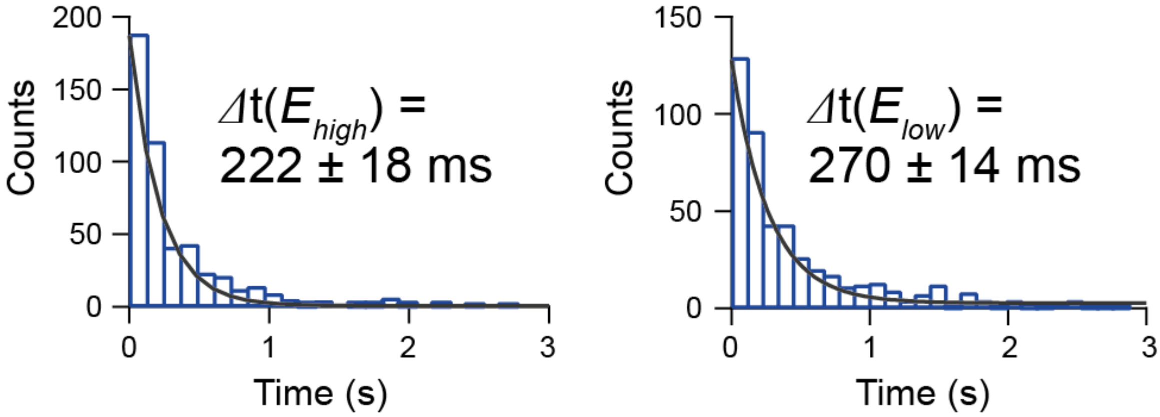

Supplementary Figure 5. Kinetic analysis of R-loop dynamics with 20-nt crRNA. The dwell time distributions of E_{high} and E_{low} with mean Δt (single exponential fit, gray line). (mean ± SEM, n ≥ 3).

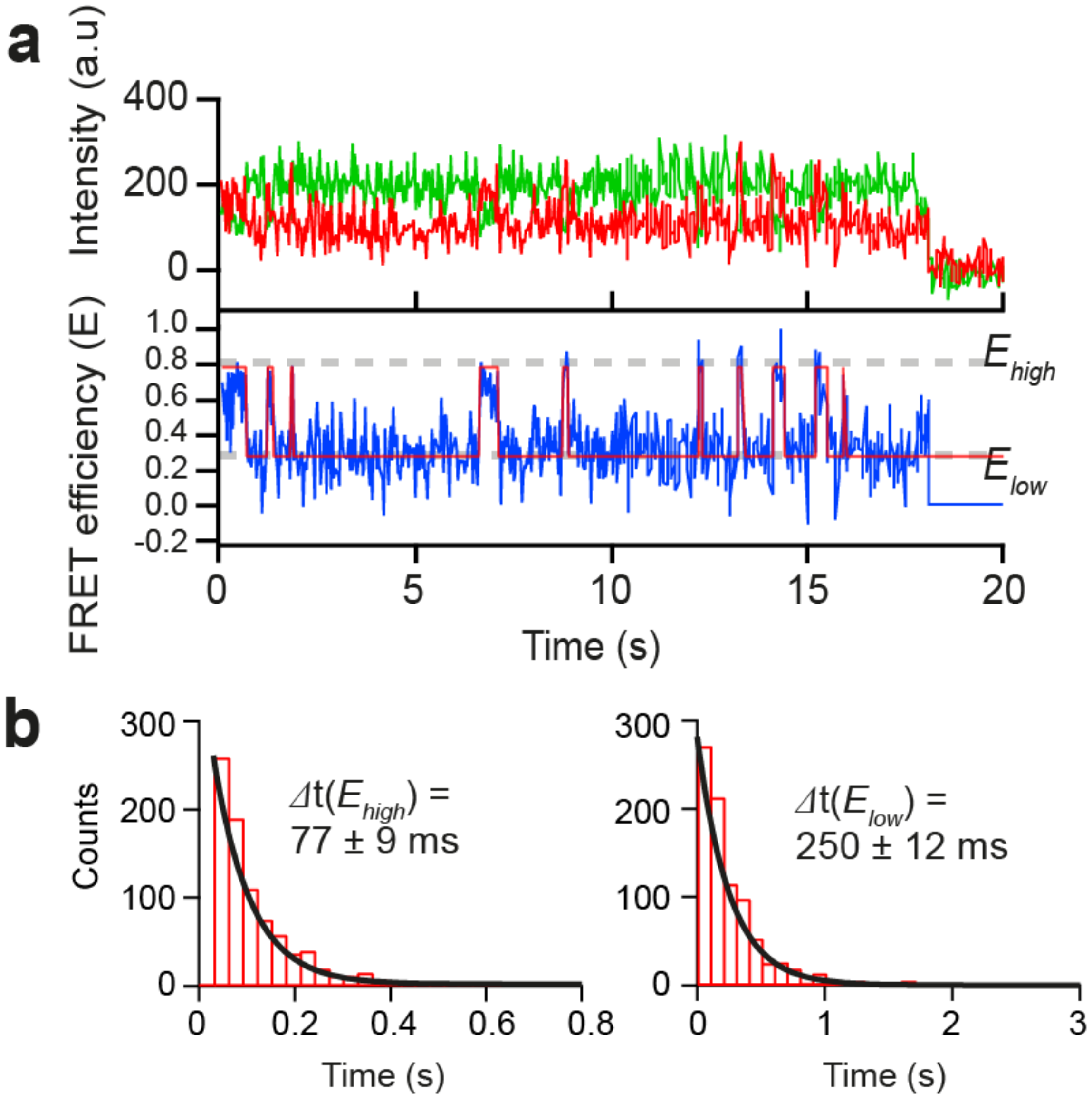

Supplementary Figure 6. Kinetic analysis of R-loop dynamics with 17-nt crRNA. (a) A representative time trajectory shows the transition of R-loop sub-conformation with applying 17-nt crRNA. The duration of each conformation is measured as the dwell time (Δt). The trajectory was imaged with an integration time of 0.03 s. In comparison with R-loop dynamics with 20-nt crRNA, the dwell time of high-FRET state became short-lived and the interface of two DNA strands at PAM-distal end is destabilized. (b) The dwell time distributions of E_{high} and E_{low} with mean Δt (single exponential fit, gray line). (mean ± SEM, n ≥ 3).

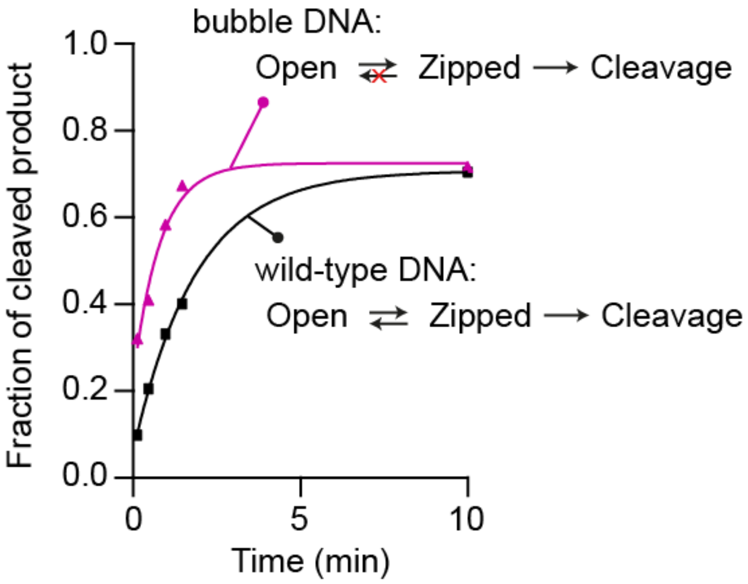

Supplementary Figure 7. Kinetic difference between the wild-type and bubble DNA. In Cas9-mediated DNA cleavage, the conformational distribution of R-loop affects the rate of DNA cleavage. The wild type exhibiting both of the open conformation and zipped conformation was cleaved slowly (black), comparing to the bubble DNA exhibiting only the zipped conformation (magenta). This indicates that the transition into open conformation is a bona fide intermediate for the formation of the zipped conformation possibly associated with the conformation for activated nuclease domain[1,2].

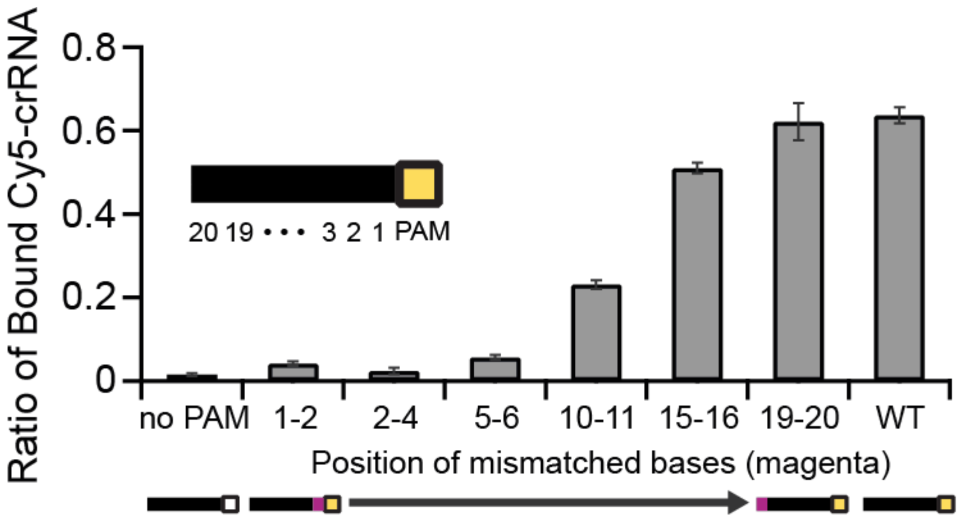

Supplementary Figure 8. Binding assay with DNA sequences incorporating mismatched bases on variable positions. The probability of DNA binding by Cas9:gRNA depends strongly on the proximity of the mismatched bases to the PAM site (mean ± SEM, n ≥ 5). Because the PAM-adjacent mismatched sequences show no significant FRET signal between the two dyes, we confirm that PAM recognition alone does not stabilize Cas9:gRNA:DNA. This result is in good agreement with previous reports that suggest an R-loop formation in the ternary complex gaining adequate stability when the target DNA helix is sufficiently unwound (~12 bp, approximately one turn of an A form RNA-DNA duplex)[3,4]. The base positions are labeled by numbering from the 5′ end of PAM. Diagrammatic representations are used for PAM (yellow square), target DNA complementary to crRNA (black bar), mismatched bases on PAM (white square), and the approximate location of mismatched bases on target DNA (magenta bar).

Supplementary Table 1. DNA and RNA sequences in this study for cleavage assay and smFRET assay.

Description	Strand	Sequences
dsDNA in cleavage assay	non-complementary	5' – /5Biosg/ttt ttt GAG GAA G/iAmMC6T/G CCT GAG TCC GAG CAG AAG AAG AAG GGC TCC CAT CAC ATC – 3'
	complementary	5' – GAT GTG A /iAmMC6T/G GGA GCC CTT CTT CTT CTG CTC GGA CTC AGG CAC TTC CTC – 3'
dsDNA in smFRET assay (WT)	non-complementary	5' – /5Biosg/ttt ttt GAG GAA G/iAmMC6T/G CCT GAG TCC GAG CAG AAG AAG AAG GGC TCC CAT CAC ATC – 3'
	complementary	5' – GAT GTG ATG GGA GCC CTT CTT CTT CTG CTC GGA CTC AGG CAC TTC CTC – 3'
no-PAM	non-complementary	5' – /5Biosg/ttt ttt GAG GAA G/iAmMC6T/G CCT GAG TCC GAG CAG AAG AAG AAC CCC TCC CAT CAC ATC – 3'
	complementary	5' – GAT GTG ATG GGA GGG GTT CTT CTT CTG CTC GGA CTC AGG CAC TTC CTC – 3'
1-2 mismatched dsDNA	non-complementary	5' – /5Biosg/ttt ttt GAG GAA G/iAmMC6T/G CCT GAG TCC GAG CAG AAG AAG TTG GGC TCC CAT CAC ATC – 3'
	complementary	5' – GAT GTG ATG GGA GCC CAA CTT CTT CTG CTC GGA CTC AGG CAC TTC CTC – 3'
2-4 mismatched dsDNA	non-complementary	5' – /5Biosg/ttt ttt GAG GAA G/iAmMC6T/G CCT GAG TCC GAG CAG AAG ATC TAG GGC TCC CAT CAC ATC – 3'
	complementary	5' – GAT GTG ATG GGA GCC CTA GAT CTT CTG CTC GGA CTC AGG CAC TTC CTC – 3'
5-6 mismatched dsDNA	non-complementary	5' – /5Biosg/ttt ttt GAG GAA G/iAmMC6T/G CCT GAG TCC GAG CAG AAC TAG AAG GGC TCC CAT CAC ATC – 3'
	complementary	5' – GAT GTG ATG GGA GCC CTT CTA GTT CTG CTC GGA CTC AGG CAC TTC CTC – 3'
10-11 mismatched dsDNA	non-complementary	5' – /5Biosg/ttt ttt GAG GAA G/iAmMC6T/G CCT GAG TCC GAG GTG AAG AAG AAG GGC TCC CAT CAC ATC – 3'
	complementary	5' – GAT GTG ATG GGA GCC CTT CTT CTT CAC CTC GGA CTC AGG CAC TTC CTC – 3'
15-16 mismatched dsDNA	non-complementary	5' – /5Biosg/ttt ttt GAG GAA G/iAmMC6T/G CCT GAG TTA GAG CAG AAG AAG AAG GGC TCC CAT CAC ATC – 3'
	complementary	5' – GAT GTG ATG GGA GCC CTT CTT CTT CTG CTC TAA CTC AGG CAC TTC CTC – 3'
19-20 mismatched dsDNA	non-complementary	5' – /5Biosg/ttt ttt GAG GAA G/iAmMC6T/G CCT CTG TCC GAG CAG AAG AAG AAG GGC TCC CAT CAC ATC – 3'
	complementary	5' – GAT GTG ATG GGA GCC CTT CTT CTT CTG CTC GGA CAG AGG CAC TTC CTC – 3'
15-20 mismatched dsDNA	non-complementary	5' – /5Biosg/ttt ttt GAG GAA G/iAmMC6T/G CCT CTC AGG GAG CAG AAG AAG AAG GGC TCC CAT CAC ATC – 3'
	complementary	5' – GAT GTG ATG GGA GCC CTT CTT CTT CTG CTC CCT GAG AGG CAC TTC CTC – 3'

crRNA	ssRNA	5' – GGG AGU CCG AGC AGA AGA AGA AGU UUU AGA GCU AUG CUG UUU UG – 3'
tracrRNA	ssRNA	5'–GGA ACC AUU CAA AAC AGC AUA GCA AGU UAA AAU AAG GCU AGU CCG UUA UCA ACU UGA AAA AGU GGC ACC GAG UCG GUG CUU UUU U– 3'
Cy5-labeled crRNA	ssRNA	5' – /5Cy5/ GAG UCC GAG CAG AAG AAG AAG UUU UAG AGC UAU GCU GUU UUG – 3'
Cy5-labled 17-bp crRNA	ssRNA	5' – /5Cy5/ UCC GAG CAG AAG AAG AAG UUU UAG AGC UAU GCU GUU UUG – 3'

"Biosg" refers to biotin, and "iAmMC6T" refers to amino-modified C6 dT linker for labeling with Cy3 or Cy5 NHS ester.

Supplementary References

1. Nishimasu, H. et al. Crystal Structure of Cas9 in Complex with Guide RNA and Target DNA. *Cell* **156**, 935-949 (2014).
2. Sternberg, S. H., LaFrance, B., Kaplan, M. & Doudna, J. A. Conformational control of DNA target cleavage by CRISPR-Cas9. *Nature* **527**, 110-113 (2015).
3. Sternberg, S. H., Redding, S., Jinek, M., Greene, E. C. & Doudna, J. A. DNA interrogation by the CRISPR RNA-guided endonuclease Cas9. *Nature* **507**, 62-67 (2014).
4. Szczelkun, M.D. et al. Direct observation of R-loop formation by single RNA-guided Cas9 and Cascade effector complexes. *Proc. Natl. Acad. Sci. USA* **111**, 9798-9803 (2014).
